# Thermal experiments with the Asian bush mosquito (*Aedes japonicus japonicus*) (Diptera: Culicidae) and implications for its distribution in Germany

**DOI:** 10.1186/s13071-018-2659-1

**Published:** 2018-02-05

**Authors:** Friederike Reuss, Andreas Wieser, Aidin Niamir, Miklós Bálint, Ulrich Kuch, Markus Pfenninger, Ruth Müller

**Affiliations:** 1 Institute for Ecology, Evolution and Diversity, Faculty of Biological Sciences, Goethe University, Max-von-Laue-Straße 9, 60438 Frankfurt am Main, Germany; 2 Senckenberg Biodiversity and Climate Research Centre, Senckenberganlage 25, 60325 Frankfurt am Main, Germany; 3 Institute of Organismic and Molecular Evolution (iOME), Faculty of Biology, Johannes Gutenberg University, Gresemundweg 2, 55128 Mainz, Germany; 4 Faculty of Medicine, Institute of Occupational Medicine, Social Medicine and Environmental Medicine, Goethe University Frankfurt, Theodor-Stern-Kai 7, 60590 Frankfurt am Main, Germany

**Keywords:** Rearing temperature, Vector mosquito, Biological invasion, Generation time

## Abstract

**Background:**

As ectothermic animals, temperature influences insects in almost every aspect. The potential disease spreading Asian bush mosquito (*Aedes japonicus japonicus*) is native to temperate East Asia but invasive in several parts of the world. We report on the previously poorly understood temperature-dependence of its life history under laboratory conditions to understand invasion processes and to model temperature niches.

**Results:**

To evaluate winter survival, eggs were exposed between 1 day and 14 days to low temperatures (5 °C, 0 °C, -5 °C and -9 °C). Hatching success was drastically decreased after exposure to 0 °C and -5 °C, and the minimal hatching success of 0% was reached at -9 °C after two days. We then exposed larvae to 14 temperatures and assessed their life trait parameters. Larval survival to adulthood was only possible between 10 °C and 31 °C. Based on this, we modelled the optimal (25 °C), minimal (7 °C) and maximal (31 °C) temperature for cumulative female survival. The time to adult emergence ranges from 12 days to 58 days depending on temperature. We used an age-at-emergence-temperature model to calculate the number of potential generations per year for the Asian bush mosquito in Germany with an average of 4.72 potential generations. At lower temperatures, individuals grew larger than at higher temperatures with female R1 length ranging from 3.04 ± 0.1 mm at 31 °C to 4.26 ± 0.2 mm at 15 °C.

**Conclusions:**

Reduced egg hatch after exposure to sub-zero temperatures prohibits the establishment of the Asian bush mosquito in large parts of Germany. Larval overwintering is not possible at temperature ≤ 5 °C. The many potential generations displayed per year may contribute to the species’ invasion success. This study on the thermal ecology of the Asian bush mosquito adds to our knowledge on the temperature dependence of the species and data could be incorporated in epidemiological and population dynamic modelling.

**Electronic supplementary material:**

The online version of this article (10.1186/s13071-018-2659-1) contains supplementary material, which is available to authorized users.

## Background

Temperature plays a major role in determining the distribution of vector mosquitoes [1] and viral transmission areas [2–4]. Climatic conditions, and especially temperature, at the point of entrance and the individuals’ temperature tolerance and population history may be key determinants for the invasion success of a mosquito. Also, the temperature can limit population growth (*Aedes albopictus*; [5]) and dispersal of an invasive mosquito species (*Ae. albopictus*; [6]) and shapes its phenology (*Aedes aegypti*; [7]). Larval rearing temperature influences development times [8–10], larval survival [8, 10, 11], adult longevity [8, 12], length of the female gonotrophic cycle [8] and adult body size [13–15] and thus fecundity [16]. In arboviruses, temperature affects plaque growth [17] and replication speed [4]. In addition, mosquito-arbovirus interactions such as virus susceptibility [4, 18, 19], prevalence of dissemination [4, 17, 19], transmission rate [4], and extrinsic incubation period [20, 21] are influenced by temperature depending on the species of mosquito and virus (reviewed in [22]).

The Asian bush mosquito (*Aedes japonicus japonicus* Theobald, 1901), a mosquito native to temperate East Asia, is a competent vector of arboviruses like West Nile virus, Japanese encephalitis virus and La Crosse virus under laboratory conditions [23–25]. The species is non-native in, among other countries, Germany where it was found for the first time in 2008 in the south-west [26]. Subsequently, the Asian bush mosquito has quickly expanded its range to parts of southern [27–29], western [30] and northern [31, 32] Germany. Knowledge of the temperature tolerance of this species is thus important for the development of epidemiological, phenological or population dynamic models to predict the further faith of these invasive populations.

Tolerance to temperature extremes determines the distribution limits in *Ae. aegypti* [7]. The egg stage is the predominant stage for overwintering in *Ae. j. japonicus* [33–35] but larval overwintering has been reported [36, 37]. However, precise data on lower thermal limits of larval hatch from eggs and survival to adulthood in the Asian bush mosquito is lacking. Also, knowledge of temperature-dependence of life history trait parameters of the Asian bush mosquito is fragmentary. The larval stage has its upper-temperature limit for development between 28 °C and 34 °C, larvae reared at 10 °C were able to emerge, and the minimum thermal temperature for larval development was calculated to 7 °C [38]. The development from egg hatch to emergence takes between 130 and 163 days at 10 °C and 11 to 18 days at 28 °C [38].

Multivoltinism likely influences the species’ evolutionary [39] and seasonal demographic dynamics [5]. Compared to other mosquito species sharing the same larval habitat, the Asian bush mosquito occurs earlier in spring [35, 36] and usually displays multiple generations per year [35, 40]. The activity period of immature stages in native Japan starts in May [36]. In Connecticut, USA, the activity period for immature stages ranges from March to November and adult females were active from June to October with highest abundances in September [40]. In New York State, USA, Asian bush mosquito females were found from May to October [41], and in Switzerland, the last observed oviposition occurred in October [42]. However, the number of potential generations per year is yet unknown and may depend on the particular environmental temperature regime in the area of occurrence.

This study presents experimental findings on the thermal biology of this invasive mosquito to explain its invasion success, potential future population developments and risks of vector-borne diseases in Germany.

## Methods

### Origin of biological material and taxonomic identification

For larval experiments, eggs of the Asian bush mosquito were collected from May to August 2015 and 2016 (Additional file 1: Table S1) using rainwater-filled black plastic buckets with inset pressboard sticks, the latter serving as an oviposition substrate. The buckets were left for seven days in a private garden in Biberach (Baden), Germany. Oviposition sticks were collected and stored in a closed plastic bag at 25 °C, 90% relative humidity and a 16:8 h light: dark photoperiod in a climate chamber (Flohr, Utrecht, the Netherlands) for at least eight days (Additional file 1: Table S1). Larval hatch was stimulated by placing oviposition sticks in deionised water at 25 °C. Only first-instar larvae younger than 24 h were used in the experiments. The day of the onset of the experiment was considered day 0. Eggs for egg experiments were collected during summer (June, July) 2016 and 2017 (Additional file 1: Table S1) to ensure that they were not in diapause. Eggs were stored in sealed plastic bags at 25 °C until experimental onset (Additional file 1: Table S1).

Unequivocal identification of eggs or larvae from *Ae. j. japonicus* is difficult without destroying them. In all experiments, larvae were therefore reared to adulthood for morphological identification to ensure the exclusive occurrence of the Asian bush mosquito: the ornamentation of the mesonotum, the colour of the fourth and fifth tarsomere and the colour of the palps were used as diagnostic characters [37].

### Egg experiments

Eggs were incubated for ten exposure periods (0, 6, 12, 18 h and 1, 2, 3, 4, 7, 14 days) at 5 °C, 0 °C, -5 °C and - 9 °C. Twenty eggs were placed on a coffee filter paper (diameter 2 cm) soaked with 200 μl deionised water and placed in a 100 ml plastic beaker. Five cups per temperature and exposure length were set up (4000 eggs in total). Larval hatch was stimulated by filling the cups with 80 ml deionised water and bringing them to 25 °C. Hatched larvae were counted on three consecutive days after hatching stimulus. Only larvae completely detached from the eggshell were counted and included when calculating the mean hatch success. Hatch success was arcsine transformed, and logistic or exponential regressions were selected based on AIC values calculated in Past 3 [43]. Based on the resulting functions, minimal hatch success was calculated.

### Life history experiments

Five 1 l cylindrical plastic cups filled with 800 ml deionised water and 40 larvae each were set up for every temperature treatment. Larval rearing temperatures were set to 0, 5, 10, 12, 14, 15, 17, 19, 20, 23, 25, 26, 27, 28, 29 and 31 °C and photoperiod to 16:8 h light: dark without crepuscular transitions. Larvae were fed with 10 mg TetraMin (Tetra, Melle, Germany) per larva in seven portions [44].

Pupae were transferred individually to 5 ml glass vials filled with deionised water. The water was removed when imagines emerged. Imagines were held in the vials without access to food or water. Ambient temperature for pupae and imagines was the same as their respective larval rearing temperature. The relative humidity in the climate chambers and rooms (Additional file 1: Table S1) was kept at 90%. The age at pupation, age at emergence, age at adult death, adult sex and mortality were recorded, and the length of the R1 wing vein was measured as described in [44]. To ensure that moribund larvae in the 0 °C treatment were dead, the cups were incubated at 10 °C for two days and then brought to 25 °C ambient temperature.

### Analysis of life trait parameters

The mean mortality in every temperature treatment was calculated in percent and arcsine transformed for statistical tests. Differences in mortality between temperature groups were tested by one-way analysis of variance (ANOVA) and pairwise two-tailed t-tests with Bonferroni-corrected *P*-values for all temperature-treatment pairs. The sex ratio was calculated as a departure from an expected 1:1 ratio with a two-sided exact binominal test. For that, the total numbers of emerged imagines summed up for the five replicates for each temperature were tested. The mean age at pupation, age at emergence and age at adult death were calculated in days. The relationship between three life trait parameters and the temperature was inferred using a nonlinear least square method. Adult body size was measured as the length of the R1 wing vein. Two-way ANOVAs were calculated to test the influence of temperature and sex on age at pupation, age at emergence, age at adult death, length of the R1 wing vein and the percentage difference to the maximal length of the R1 wing vein.

### Modelling of limiting and optimal temperatures

The product of the developmental rate of females (inverse age at emergence) and survival was calculated as a measure of thermal performance (cumulative female survival). Model selection and calculation of minimum and maximum temperatures for development were done by testing all models implemented in the R github package *thermPerf* [45]. The package was used for exploratory purposes only, and the actual fit was manually made in R. The optimal temperature for cumulative female survival was calculated according to Briere et al. [46] (eq. 3).

### Estimation of the potential number of generations per year

Exponential curves were fitted to the mean age at pupation, age at emergence and age at adult death large-scale to temperature. The age at an emergence-temperature function (Additional file 1: Table S3) was adjusted by 14 days to estimate the generation time as we found that eggs were laid two days after an offered artificial blood meal and about 14 days after adult emergence in the laboratory in the studied population (unpublished data). However, oviposition only occurred once, and eggs did not hatch (for possible reasons see [47, 48]).

The potential number of generations per year in Germany was estimated for present climatic conditions and future climate projections. For the present condition, the function for generation time was fitted to the mean monthly temperatures for Germany at a resolution of 2.5′ (WorldClim database [49]). We calculated the number of generations per day and the cumulative number of potential generations per year. For future climate projections, the monthly average maximum and a minimum temperature of the CCSM4 model with a low and high greenhouse gas scenario, RCP2.6 and RCP8.5, for the period 2041 to 2050 was used (WorldClim database). As a final result, the minimum and maximum numbers of potential generations in Germany were extracted from the parameterised layer. As examples, six points of occurrence in Germany for the Asian bush mosquito were taken from the literature [26, 29, 31, 44, 50] (Additional file 1: Table S4) and the numbers of potential generations were calculated for these locations.

All calculations were made in R3.1.1 [51] via RStudio 1.0.136 [52] with the following R packages and their respective dependencies: *sp.* [53, 54], *raster* [55] and *rgdal* [56] for spatial data handling and visualisation and *rasterVis* [57] for raster data visualisation. The package *thermPerf* [45] and its prerequisite *devtools* [58] were used for model selection of the thermal performance curve.

## Results

### Eggs as the overwintering stage

**Fig. 1:**
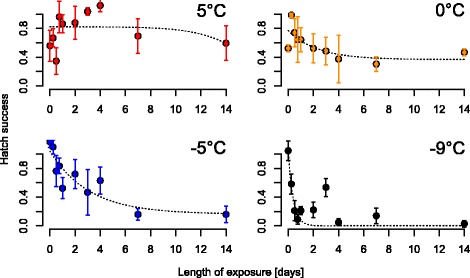
Larval hatch success of eggs exposed to 5 °C, 0 °C, -5 °C and -9 °C for up to 14 days. Hatch success represents the cumulative proportion of hatched larvae counted at three consecutive days after hatch stimulus

Egg incubation at temperatures below 5 °C resulted in strongly reduced larval hatching success (Fig. 1). Larval hatch success after incubation at 5 °C was not significantly affected by exposure length (*F*_(1,8)_ = 0.02, *P* = 0.9). Exponential or logistic curves were fitted to the datasets for 0 °C, -5 °C and -9 °C, which showed an exposure length-dependent trend. The curves are best described by the functions:

y_0 °C_ = 32.94 exp.(-0.41×) + 36.12,

y_-5 °C_ = 74.01 exp.(-0.22×) + 10.67 and

y_-9 °C_ = 7.56 × 10^8^/ (1 + 7.25 × 10^8^ exp.(2.5621×)).

Thus, the minimal hatching success was 36% at 0 °C, 10% at -5 °C and 0% at -9 °C.

### The effect of temperature on the cumulative larval and pupal mortality and development

**Fig. 2:**
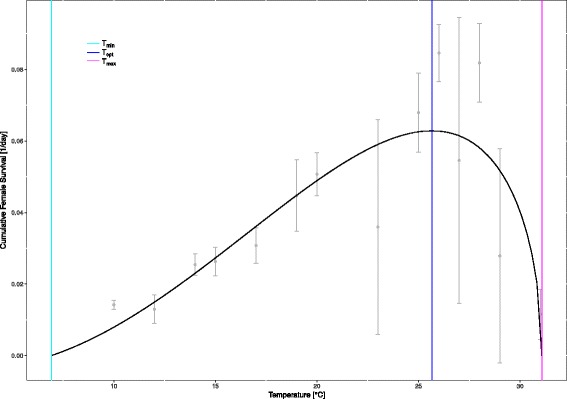
Thermal reaction norm and limits for the development and survival of the Asian bush mosquito. Cumulative female survival per day assessed over a range of 14 constant temperatures is shown. The solid black line represents the fitted curve calculated with Briere et al. [46] eq. 1. The function has the following form: cumulative female survival *y = aT(T-T*_*min*_*)(T*_*max*_*-T)*^*1/m*^ with a, m: empirical constants (determined during model selection; Additional file 1: Figure S2), *Abbreviations*: T, temperature; T_min_, minimum temperature for cumulative female survival; T_max_, maximum temperature for cumulative female survival; T_opt_, optimum temperature for cumulative female survival calculated with the first derivative equation dy/dT = 0

Overall, the temperature did significantly affect mortality (*F*_(1,98)_ = 9.90, *P* = 0.002). No adult mosquitoes emerged at the two lowest temperatures (0 °C, 5 °C; Table 1). Larvae survived for at most three days at 0 °C. Larvae reared at 5 °C almost completely died and eventually, only one survived in larval stage > 100 days. In all other temperature treatments, the mean mortality was ≤ 50% (Table 1). Cumulative female survival was calculated and upper and lower estimated developmental thresholds were provided (Fig. 2). The best fit of temperature-dependent cumulative female survival was Briere et al. [46] eq. 1-model (Additional file 1: Figure S2) with a = 6 × 10^−6^ and m = 2.0. The model selection based on AIC weights is presented in Additional file 1: Figure S2. Optimum temperature was 26 °C and lower and upper thermal limits were 7 °C and 31 °C, respectively (Table 1, Additional file 1: Figure S1).

**Table 1:** Temperature effects on mortality of the Asian bush mosquito. Mean mortality for each temperature treatment

Temperature (°C)	Mean mortality (%)	SD (%)
0	100.0	0.0
5^a^	99.5	1.1
10	16.0	5.5
12	38.5	14.2
14	18.0	4.8
15	15.0	7.9
17	19.0	9.6
19	29.5	12.4
20	11.3	6.5
23	48.5	27.6
25	13.8	8.4
26	6.0	5.2
27	41.5	31.1
28	12.5	7.7
29	70.5	22.2
31	87.5	6.4

^a^For 5 °C, the mortality was calculated on an experimental day 113. One individual survived to this day and was considered alive in the analysis. For all other temperatures, mortality reflects cumulative larval and pupal mortality

*Abbreviation*: *SD* standard deviation

Age at pupation, emergence and adult death decreased with increasing temperature (Additional file 1: Figure S3). The first pupation occurred in males on day eight at 26 °C to 29 °C. One day later, females started to pupate. At the lowest experimental temperature where emergence occurred (10 °C), pupation started later than at day 40 (Additional file 1: Table S2). Age at pupation and age at emergence were significantly affected by temperature treatment (*F*_pup(3,2466)_ = 11,043.6, *P*_pup_ ≤ 0.001; *F*_em(3,2466)_ = 10,448.6, *P*_em_ ≤ 0.001) and sex (*F*_pup(3,2466)_ = 27.8, *P*_pup_ ≤ 0.001; *F*_em(3,2466)_ = 14.2, *P*_em_ ≤ 0.001) while age at adult death was significantly affected by sex (*F*_(3,2457)_ = 53.7, *P* ≤ 0.001), temperature (*F*_(3,2457)_ = 12,614.0, *P* ≤ 0.001) and their interaction (*F*_(3,2457)_ = 14.4, *P* ≤ 0.001). For all three parameters, temperature explained more than 80% of the variance. With these data, we estimated the generation time with a nonlinear model to the age of emergence at different temperatures:

G = 290.75 exp.(-0.17 × T) + 22.32 (Additional file 1: Table S3, Additional file 1: Figure S3).

where G is the number of potential generations per year and T is the temperature (°C).

### Effect of temperature on body size and sex ratio

An exact binominal test shows a deviation from a balanced (1:1) sex ratio of 15 °C, 27 °C and 31 °C (Additional file 1: Table S5). For all other temperature treatments, the sex ratio did not significantly differ from being balanced. However, overall slightly more females than males emerged (Additional file 1: Table S5).

**Fig. 3:**
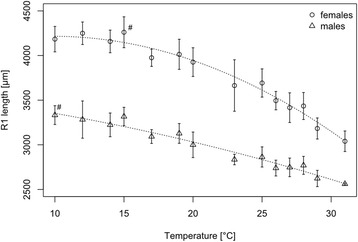
Influence of temperature and sex on the body size. R1 wing vein length in dependence of temperature. Means ± standard deviations are shown. #: Maxima of R1 wing vein length

The Asian bush mosquito showed sexual size dimorphism with females growing larger than males, taking the length of the R1 wing vein as a proxy for body size. This was valid for all temperatures for which individuals reached the adult stage (Fig. 3). Temperature (*F*_(2,25)_ = 54.4, *P* < 0.005) and sex (*F*_(2,25)_ = 622.5, *P* < 0.001) were significant sources of variation. However, analysis of the percentage difference to the maximal length of the R1 wing vein showed temperature as significant source of variation (*F*_(3,24)_ = 325.4, *P* < 0.001) but not sex or their interaction. Thus, temperature treatments influenced male and female body size similarly.

### Larvae as overwintering stages

**Fig. 4:**
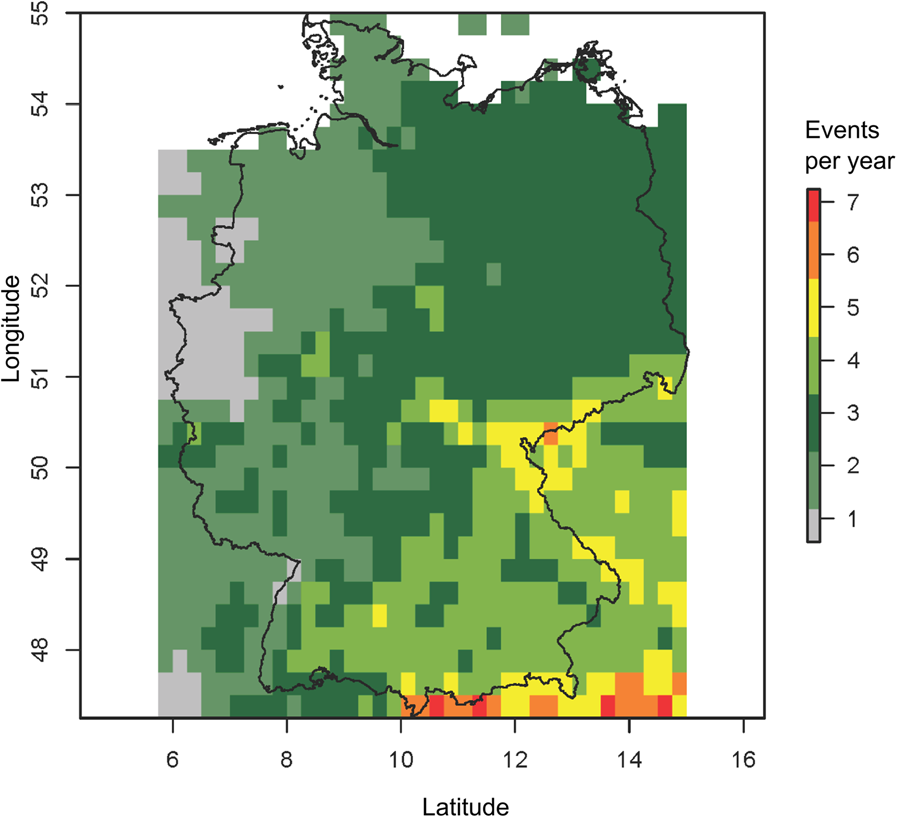
Average number of frost events with three days or longer at 0 °C or lower temperatures per year (1950–2016) in Germany. The temperature threshold was experimentally assessed and is valid for the larval stage, i.e. larvae do not survive such frost events

We analysed the daily high-resolution gridded dataset of surface temperature (E-OBS version 14.0; http://ensembles-eu.metoffice.com) provided by the ENSEMBLES project in the period from 1 January 1950 to 31 August 2016 [59] under the assumption that larvae do not survive longer than three days at 0 °C or lower temperatures (see Results - The effect of temperature on the cumulative larval and pupal mortality and development). These specific conditions have occurred on average from 1 to 6 events per year during the above mentioned period in Germany, with an increase to the south-east (Fig. 4).

### Potential number of generations per year

**Fig. 5:**
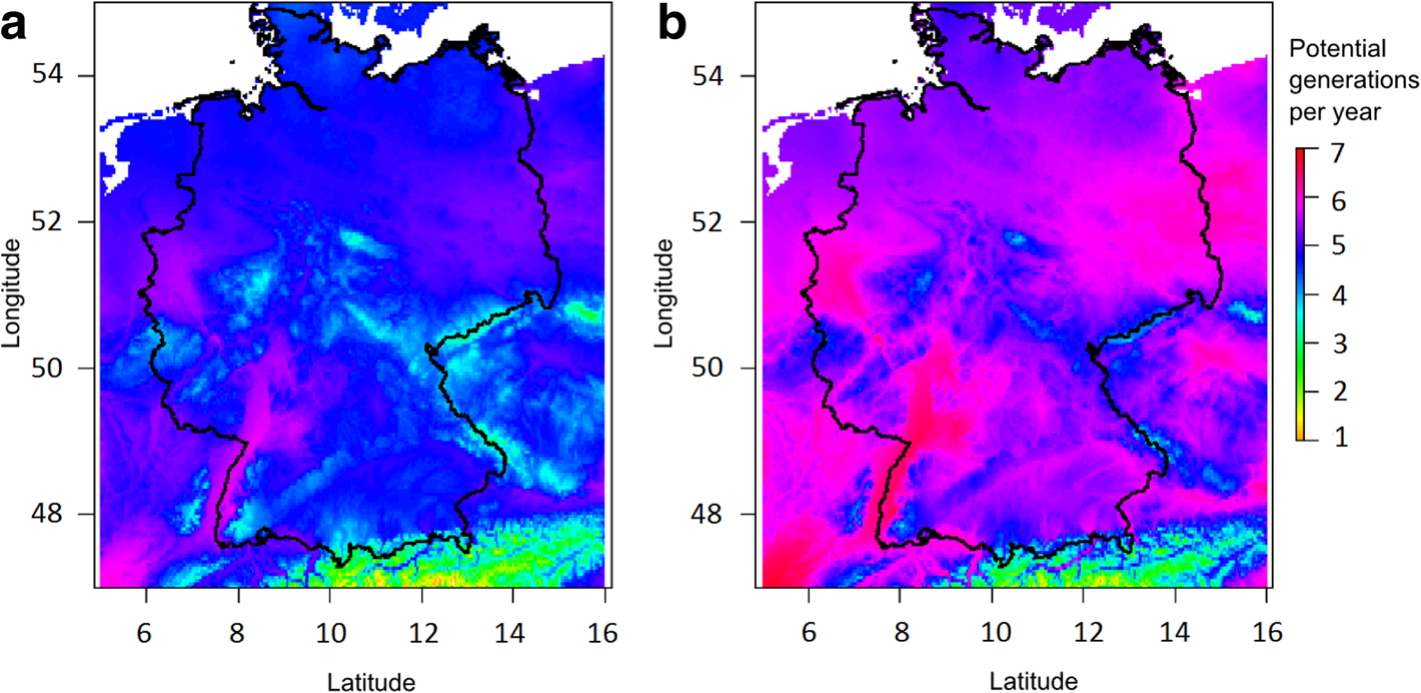
Number of potential generations of the Asian bush mosquito in Germany. Estimation of the number of potential generations per year (**a**) at present and (**b**) in the future (CCSM4; 2041 to 2060) with a low greenhouse gas emission model (RCP2.6)

The mean number of potential generations in Germany was 4.72 (range 1.92–5.77) for present climate conditions (Fig. 5a). For six locations (Additional file 1: Table S4), at which the occurrence of the Asian bush mosquito is confirmed, the potential number of generations ranged between 4.62 and 5.60 potential generations per year (Fig. 5a). There were no regions with equal or less than one potential generation per year in Germany and only in a small part of the Alps (Berchtesgadener Land), two or less potential generations per year were suggested by the model.

For the future scenario with low greenhouse gas emission, the mean number of potential generations per year increased to 5.47 (range 2.43–6.57; Fig. 5b). Under this scenario, there will be no region in Germany where two or less potential generations per year occur. The potentially least affected areas with three or fewer generations are the Alpine regions of Oberallgäu and Berchtesgadener Land. The same global change model with high greenhouse gas emissions showed on average 5.75 potential generations per year (range 2.74–6.97) for Germany.

## Discussion

Our laboratory experiments contribute to our understanding of mosquito thermal biology, which determines ecological and epidemiological features such as invasion success of a disease vector. The Asian bush mosquito can develop to female adults in temperatures ranging from 7 °C to 31 °C. Albeit the higher temperature limits of the species are not reached in Germany on a mean monthly basis, lower temperature limits of eggs and larvae seem to determine the species’ distribution. Also, we present temperature-dependent life trait data.

### Temperature dependence of life trait parameters

As expected, all tested life history parameters of the Asian bush mosquito depended strongly on ambient temperature. The minimum thermal temperature for development of the Asian bush mosquito from the first-instar to adult collected in USA was calculated between 12.6 °C (females) and 9.4 °C (males; [38]) while our estimates yielded 7 °C as the lower threshold of developmental temperature. Differences may be explained with differently used regression methods while a biological explanation maybe post-invasive local adaptation or a different origin of the respective invasive populations. The optimum temperature presented here matches the optimal temperature known for laboratory rearing of this species (25 °C; [47, 48]). The upper threshold lies between 28 °C and 34 °C for a North American population [38]. This range covers the upper-temperature threshold for development calculated here. At present, the Asian bush mosquito occurs in places with mean monthly temperatures between -2.7 °C and 11.0 °C in Germany [50]. Here, we show that individuals exposed to constant ambient temperatures between 10 °C and 31 °C reach the adult stage, while larvae cannot survive 0 °C and 5 °C.

The overall dataset yielded a balanced ratio albeit with exceptions which can be considered statistic artefacts. The experiments show that growth in this species follows the temperature-size rule for ectotherms which states that individuals grow slower at lower temperatures and become larger in body size. This holds true for both sexes (Additional file 1: Table S2, Fig. 3) and could be a reproductive strategy to compensate longer development times with an increased number of offspring. The female body size was shown to be an accurate predictor of the fecundity of two aedine mosquitoes, *Ae. albopictus* and *Aedes geniculatus*, with increasing number of mature follicles with increasing wing lengths [16]. For La Crosse virus infection, it was shown that female wing length of the Asian bush mosquito has no significant effect on the infection status [60]. The Asian bush mosquito is considered to be a minor vector for arbovirus transmission, and no wild-caught adult female was found to carry pathogens so far in Germany. Under laboratory conditions, however, the Asian bush mosquito was found to transmit arboviruses causing West Nile fever or Japanese encephalitis. For aedine mosquitoes, it was shown that mosquito-arbovirus interactions such as infection and transmission rate or extrinsic incubation period depend on temperature as well as temperature variation. However, the nature of the temperature-dependence is mosquito-specific and virus-specific [22].

Compared to *Culex pipiens* (*sensu stricto*) and *Culex quinquefasciatus*, the survival at 10 °C is much higher in the Asian bush mosquito (50% survival versus 85% survival; [61]), but the time to adult emergence is much slower in the Asian bush mosquito at temperatures between 10 °C and 25 °C [61]. In this study, the larvae were 0 to 24 h old at the onset of the experiment. Thus, the age at moulting and adult death may be slightly underestimated for the Asian bush mosquito.

The Asian bush mosquito is known to be multivoltine in Japan and North America [34, 38]. Immature and mature stages are found at the same time in the same place. Thus, it is assumed that overlapping generations exist [36, 40]. We confirm this observation at our sampling site. Our model to calculate the number of potential generations per year is based on the correlation between the age at emergence and the temperature correlation. We shifted up this function by 14 days to account for copulation, blood meal consumption and egg production. There is evidence that eggs have to mature for 12 to 17 days after oviposition [47]. Williges et al. [47] and Hoshino et al. [48] report 28 generations in eight years (3.5 generations per year), respectively, 35 generations in five years (7 generations per year) for laboratory colonies of the Asian bush mosquito maintained at 25 °C. To our knowledge, no estimates for the potential number of generations in field populations are reported yet.

### Temperature limits to the distribution in Germany

Today, the Asian bush mosquito is known to occur in southwestern Germany (Baden-Württemberg; [26, 27, 42]), in western Germany (Rhineland-Palatinate, North Rhine Westphalia; [29, 30, 50]) and the southern part of Lower Saxony [31]. Interestingly, in the area with the least potential generations per year in the present climatic conditions (southern Germany, Berchtesgaden), the Asian bush mosquito was found in 2015 [62]. In Germany, mean monthly temperatures, as an approximation for relevant water temperatures, do not reach the upper thermal limit for cumulative female survival (Fig. 2) and also the optimal temperature for development is not reached. However, the lower temperature limit is limiting (Figs. 2 and 4). Thus, population growth is restricted by temperature. Based on our data, we hypothesise that the population in Berchtesgaden will not persist multi-annually without migration or human-assisted re-introduction.

### Overwintering

Our findings of the lower limit of lethal temperatures of 7 °C for larvae coincides with published calculations of the minimum thermal temperature for larval development of 7 °C [38]. Scott [38] showed that no male Asian bush mosquitoes and only 50% females of a New Jersey, USA, based laboratory colony pupated at 10 °C. Here, we show that individuals of both sexes of a German population can reach the adult stage at this temperature. We also could show that some larvae could survive for up to three days at 0 °C as first-instars. Thus, it is possible that both sexes of the Asian bush mosquito can temporarily survive low temperatures in the larval stage. In Japan, the Asian bush mosquito overwinters in the larval stage [37], but outside its native range, no larval overwintering was observed yet. Our results show that larval overwintering in Germany is not possible because at least one frost event occurs (Fig. 4). However, under suitable microclimatic conditions, e.g. in anthropogenic microhabitats in cities, larval winter survival may be possible, especially in western Germany.

Larval hatching success is strongly reduced after exposure to and below 0 °C. This means that for most of Germany, one may regularly expect a strong population reduction under the prevailing winter conditions. Population survival is prohibited by prolonged, severe frost periods. However, repeated frost-thaw cycles were not tested and may further reduce winter survival of populations.

Under climate change condition, the number of frost events may be reduced and the number of generations per year may be increased which increase population growth and invasion speed.

### Invasion success

Compared to other container breeding mosquitoes, the Asian bush mosquito is found in water-filled containers which show a cooler water temperature and that are partially or fully shaded [63]. This is reflected by eq. 1 in Briere et al. [46] which we found to be the best model to explain larval performance in this species. This model shows exponential growth of cumulative female survival at low temperatures which increases until the optimum temperature and sharply decreases to the lethal thermal maximum temperature. In addition, adult females are found in higher abundances in autumn than in summer [40], the proportion of immature stages of the Asian bush mosquito is higher in October than in September compared to the Asian tiger mosquito *Ae. albopictus* [63] and adult as well as immature stages show broader seasonal activity periods than other species of *Aedes*/*Ochlerotatus* [41]. Its phenology of rapid population increase in early spring may hold a developmental advantage over other container-inhabiting mosquitoes [35, 64].

Despite the prevailing winter conditions, thermal conditions allow at least one generation per year, in most areas more (Fig. 5). However, the build-up of large populations requires several generations per year, which occurs rather in the areas of also permissible winter conditions. Conversely, the areas with the strongest winter reduction are also the areas, where only one or two generations per year are possible, which may in sum prevent a successful, stable colonisation.

In (sub-)tropical invasive mosquitoes, e.g. the Asian tiger mosquito, the temperature is the dominant factor limiting the species’ distribution in mountainous regions [6]. In the temperate Asian bush mosquito, temperature also limits the distribution as pointed out here and temperature-dependent development data may be included in species’ distribution modelling. Besides the Asian bush mosquito, *Aedes koreicus* is also a temperate mosquito species which arrived in Germany [65]. This further introduction highlights the importance of studying ecological and physiological features of temperate mosquitoes to allow predictions on key risk areas and future distributions.

### Limitations of this study

The successful establishment of an exotic species in a newly colonised region is not only dependent on temperature. Factors like availability of unoccupied ecological niches and avoidance of competition due to temporal segregation of oviposition [35, 64] were hypothesised to play a role in the Asian bush mosquito’s invasion success. Also, the colonisation dynamics of *Ae. j. japonicus* on the over-regional scale likely depends on passive transport which is thought to play a major role in its long-distance dispersal as inferred by genetic population structure [32]. While probably not relevant for the demographic dynamics of an established species, inherently unpredictable passive transport may play a crucial role for initial colonisations and the sudden and ephemeral occurrence of populations (e.g. Berchtesgaden in [62]). Information on the dispersal mode, other non-thermal factors, and their impact on distribution need to be integrated into predictions of the occurrence of *Ae. j. japonicus*. However, thermal conditions set the limits of possible occurrence and thus determine the probability of long-term population establishment.

In our experiments, mosquitoes were exposed to constant temperatures during larval, pupal and adult life stages. Therefore, we did not account for (diurnal or seasonal) temperature fluctuations which are experienced by natural populations and may affect the assessed life trait parameters. Also, it must be noted that we studied one population only. Genetic data suggest that the populations in Germany show population sub-structuring due to population admixture by active migration and repeated passive introductions [29, 32, 66]. Thus, local adaptation to temperature may vary among local populations and geographical regions. Thus, the generalisation of the data is difficult. That is why we focused our analyses on Germany only. For egg experiments, eggs collected in summer were used (Additional file 1: Table S1) thus presented results rely on non-diapausing eggs.

## Conclusions

The studied population of the Asian bush mosquito has a broad temperature range (7 °C to 31 °C) suitable for growing to adult females; the optimal temperature was calculated as 26 °C. The studied population displays up to six potential generations per year in Germany. According to our data, large scale larval overwintering is not possible. The temperature limit for eggs is -9 °C for two days or longer. The broad temperature range for development may have contributed to the invasion success of the species in Germany while population establishment may not be possible in regions with low numbers of generations per year and a high number of frost events for larvae and eggs as defined here.

## Additional file

Additional file 1: Table S1. Dates of egg collections and experimental onsets. **Table S2.** Age at pupation, age at emergence and age at adult death analysed for males and females. **Table S3.** Exponential curve parameters to estimate the generation time. The exponential curves are described as following: y = a × exp.(−bx) + c. The estimate of the duration of one generation is the age at emergence plus shifted 14 days up for taking blood meal and reproduction into account [offset c + 14 (days)]. **Table S4.** Selection of localities of present occurrence and number of potential generations per year. **Table S5.** Test statistics for sex ratio tests. **Table S6.** R1 wing vein length measurements. **Figure S1.** Median mortality per temperature. Boxplots show quartile ranges and medians and whiskers depict 1.5 times the interquartile range. Mortality was calculated considering individuals, which did not survive to emergence. Mortality of the 5 °C experiment was assessed on day 113 (see Table 1). **Figure S2.** Thermal performance: results of model selection. Model selection using the github R package *thermPerf* with the cumulative larval survival as performance variable. A: The importance of each model is shown as AIC weights with low weights showing models better fitting to the data. B: Fits of all ten models with the best fit as green line. During model selection, the empirical constants were calculated to a = 0.00006 and m = 2.127 for Briere et al. [46] eq. 2. Since m = 2 is implemented in Briere et al. [46] eq. 1 and since this model was considered best for fitting the data, non-linear regression of temperature-dependent cumulative female survival was done with this model (R^2^_adj_ = 0.68) in order to calculate minimum, optimum and maximum temperatures for cumulative female survival. **Figure S3.** Life trait parameters as functions of temperature. Exponential curves fitted to the life cycle parameters age at pupation, age at emergence and age at adult death. The curve parameters are given in Table S3. (DOCX 112 kb)

## Abbreviations

AIC: Akaike information criterion
ANOVA: Analysis of variance
G: Number of potential generations per year
SD: Standard deviation
T: Temperature
T_max_: Maximum temperature for cumulative female survival
T_min_: Minimum temperature for cumulative female survival
T_opt_: Optimum temperature for cumulative female survival
