# Supplementary material for: Thermal experiments with the Asian bush mosquito (Aedes japonicus japonicus) (Diptera: Culicidae) and implications for its distribution in Germany
Source: Parasit Vectors. 2018 Feb 5;11:81. doi: 10.1186/s13071-018-2659-1 (PMC5800082; doi:10.1186/s13071-018-2659-1)
Supplement: Additional file 1: Table S1. — Dates of egg collections and experimental onsets. Table S2. Age at pupation, age at emergence and age at adult death analysed for males and females. Table S3. Exponential curve parameters to estimate the generation time. The exponential curves are described as following: y = a × exp.(−bx) + c. The estimate of the duration of one generation is the age at emergence plus shifted 14 days up for taking blood meal and reproduction into account [offset c + 14 (days)]. Table S4. Selection of localities of present occurrence and number of potential generations per year. Table S5. Test statistics for sex ratio tests. Table S6. R1 wing vein length measurements. Figure S1. Median mortality per temperature. Boxplots show quartile ranges and medians and whiskers depict 1.5 times the interquartile range. Mortality was calculated considering individuals, which did not survive to emergence. Mortality of the 5 °C experiment was assessed on day 113 (see Table 1). Figure S2. Thermal performance: results of model selection. Model selection using the github R package thermPerf with the cumulative larval survival as performance variable. A: The importance of each model is shown as AIC weights with low weights showing models better fitting to the data. B: Fits of all ten models with the best fit as green line. During model selection, the empirical constants were calculated to a = 0.00006 and m = 2.127 for Briere et al. [46] eq. 2. Since m = 2 is implemented in Briere et al. [46] eq. 1 and since this model was considered best for fitting the data, non-linear regression of temperature-dependent cumulative female survival was done with this model (R2adj = 0.68) in order to calculate minimum, optimum and maximum temperatures for cumulative female survival. Figure S3. Life trait parameters as functions of temperature. Exponential curves fitted to the life cycle parameters age at pupation, age at emergence and age at adult death. The curve parameters are given in Table S3. (DOCX 112 kb) [file 13071_2018_2659_MOESM1_ESM.docx]

**Additional file 1**

**Table S1.** Dates of egg collections and experimental onsets. **Table S2.** Age at pupation, age at emergence and age at adult death analysed for males and females. **Table S3.** Exponential curve parameters to estimate the generation time. The exponential curves are described as following: y = a × exp(-bx) + c. The estimate of the duration of one generation is the age at emergence plus shifted 14 days up for taking blood meal and reproduction into account [offset c+14 (days)]. **Table S4.** Selection of localities of present occurrence and number of potential generations per year. **Table S5.** Test statistics for sex ratio tests. **Table S6.** R1 wing vein length measurements. **Figure S1.** Median mortality per temperature. Boxplots show quartile ranges and medians and whiskers depict 1.5 times the interquartile range. Mortality was calculated considering individuals, which did not survive to emergence. **Figure S2.** Thermal performance: results of model selection. Model selection using the github R package *thermPerf* with the cumulative larval survival as performance variable. A: The importance of each model is shown as AIC weights with low weights showing models better fitting to the data. B: Fits of all ten models with the best fit as green line. **Figure S3.** Life trait parameters as functions of temperature. Exponential curves fitted to the life cycle parameters age at pupation, age at emergence and age at adult death. The curve parameters are given in Table S3.

**Additional file 1: Table S1.** Dates of egg collections and experimental onsets.

| Temperature [°C] | Date of egg collection | Date of experimental  onset (day 0) | Climate chamber |
| --- | --- | --- | --- |
| 0 | 07+14-Aug-2016 (pooled) | 23-Aug-2016 | Rumed^a^ |
| 5 | 07+14-Aug-2016 (pooled) | 23-Aug-2016 | Rumed^a^ |
| 10 | 07+14-Aug-2016 (pooled) | 23-Aug-2016 | Rumed^a^ |
| 12 | 23-May-2015 | 31-May-2015 | Flohr^b^ |
| 14 | 23-May-2015 | 31-May-2015 | Flohr^b^ |
| 15 | 23-May-2015 | 31-May-2015 | Flohr^b^ |
| 17 | 26-Jun-2015 | 30-Jun-2015 | Rumed^a^ |
| 19 | 21-Jul-2015 | 27-Aug-2015 | Rumed^a^ |
| 20 | 23-May-2015  Repeat: 23-May-2015 | 31-May-2015  Repeat: 31-May-2015 | Flohr^b^  Repeat: MH^c^ |
| 23 | 26-Jun-2015 | 30-Jun-2015 | MH^c^ |
| 25 | 23-May-2015  Repeat: 26-Jun-2015 | 31-May-2015  Repeat: 17-Jul-2015 | Flohr^b^  Repeat: Flohr^b^ |
| 26 | 23-May-2015 | 31-May-2015 | MH^c^ |
| 27 | 26-Jun-2015  Repeat: 21-Jul-2015 | 30-Jun-2015  Repeat: 10-Aug-2015 | Rumed^a^  Repeat: Flohr^b^ |
| 28 | 4-Jul-2016 | 11-Jul-2016 | Rumed^a^ |
| 29 | 26-Jun-2015  Repeat: 21-Jul-2015 | 30-Jun-2015  Repeat: 10-Aug-2015 | Rumed^a^  Repeat: white^d^ |
| 31 | 15-Jun-2015 | 22-Jun-2015 | Flohr^b^ |
| 5 (eggs) | 26-Jul-2016 | 08-Aug-2016 | Rumed^a^ |
| 0 (eggs) | 21-Jul-2016 | 26-Jul-2016 | Rumed^a^ |
| -5 (eggs) | 03-Jun-2017 | 10-Jun-2017 | Rumed^a^ |
| -9 (eggs) | 21-Jul-2016 | 26-Jul-2016 | Liebherr^e^ |

^a^: Rumed 304; Rubarth Apparate, Laatzen, Germany. ^b^: MKKL 1200; Flohr, Utrecht, the Netherlands. ^c^: Senckenberg BiK-F mesocosm hall. ^d^: Heraeus BK 600; Kendro, Hanau, Germany. ^e^: GGU; Liebherr, Biberach a. d. Riß, Germany.

**Additional file 1: Table S2.** Age at pupation, age at emergence and age at adult death analysed for males and females.

| Temperature [°C] | Age at pupation  ± sd [days] | | Age at emergence  ± sd [days] | Age at adult death  ± sd [days] |
| --- | --- | --- | --- | --- |
| Males | | | | |
| 10 | 43.1 ± 2.5 |  | 57.5 ± 2.5 | 67.1 ± 5.0 |
| 12 | 35.9 ± 2.6 | | 46.0 ± 2.6 | 54.8 ± 5.3 |
| 14 | 23.9 ± 2.1 | | 30.7 ± 2.1 | 40.1 ± 3.9 |
| 15 | 25.2 ± 2.0 | | 31.1 ± 2.1 | 40.1 ± 3.0 |
| 17 | 20.8 ± 2.1 | | 25.4 ± 2.1 | 33.5 ± 2.2 |
| 19 | 11.8 ± 0.9 | | 15.0 ± 0.9 | 21.0 ± 2.6 |
| 20 | 13.0 ± 1.2 | | 16.3 ± 1.3 | 22.8 ± 2.2 |
| 23 | 10.5 ± 0.9 | | 13.3 ± 1.2 | 18.6 ± 1.5 |
| 25 | 9.7 ± 1.1 | | 12.0 ± 1.2 | 17.2 ± 1.5 |
| 26 | 8.4 ± 0.6 | | 10.7 ± 0.6 | 15.8 ± 1.8 |
| 27 | 8.2 ± 1.0 | | 10.5 ± 1.3 | 15.1 ± 2.0 |
| 28 | 8.0 ± 0.8 | | 10.3 ± 0.6 | 15.3 ± 1.5 |
| 29 | 8.0 ± 0.8 | | 10.0 ± 0.9 | 12.4 ± 1.6 |
| 31 | 11.0 ± 1.4 | | 12.5 ± 2.1 | 15.0 ± 1.4 |
| Females | | | | |
| 10 | 45.1 ± 3.0 | | 59.3 ± 3.1 | 73.3 ± 3.8 |
| 12 | 37.9 ± 3.1 | | 47.8 ± 3.2 | 59.5 ± 3.9 |
| 14 | 25.6 ± 2.2 | | 32.4 ± 2.2 | 42.7 ± 3.6 |
| 15 | 26.1 ± 2.3 | | 32.1 ± 2.4 | 42.4 ± 2.5 |
| 17 | 21.4 ± 1.7 | | 26.2 ± 1.8 | 34.5 ± 2.0 |
| 19 | 12.8 ± 1.2 | | 15.8 ± 1.4 | 22.8 ± 3.0 |
| 20 | 14.0 ± 1.3 | | 17.5 ± 1.5 | 26.0 ± 2.4 |
| 23 | 11.0 ± 0.8 | | 13.9 ± 0.9 | 19.5 ± 2.0 |
| 25 | 10.2 ± 1.2 | | 12.6 ± 1.3 | 18.3 ± 1.6 |
| 26 | 8.8 ± 0.7 | | 11.1 ± 0.7 | 16.8 ± 1.0 |
| 27 | 8.8 ± 1.1 | | 11.0 ± 1.2 | 15.8 ± 1.8 |
| 28 | 8.7 ± 1.0 | | 10.8 ± 0.9 | 16.3 ± 1.6 |
| 29 | 8.8 ± 1.0 | | 10.8 ± 1.1 | 13.5 ± 1.7 |
| 31 | 9.7 ± 1.2 | | 11.2 ± 1.3 | 14.4 ± 1.3 |

**Additional file 1: Table S3.** Exponential curve parameters to estimate the generation time. The exponential curves are described as following: y = a × exp(-bx) + c. The estimate of the duration of one generation is the age at emergence plus shifted 14 days up for taking blood meal and reproduction into account [offset c+14 (days)].

| Life trait parameter | a [days]  (95% CI) | b [1/°C]  (95% CI) | c [days]  (95% CI) | R^2^ |
| --- | --- | --- | --- | --- |
| Age at pupation | 200.70  (121.61-279.79) | 0.16  (0.12-0.20) | 6.48  (3.85-9.11) | 0.87 |
| Age at emergence | 290.75  (199.09-382.40) | 0.17  (0.14-0.20) | 8.32  (5.89-10.76) | 0.92 |
| Age at adult death | 272.42  (199.97-344.85) | 0.15  (0.12-0.17) | 10.91  (7.73-14.09) | 0.95 |

**Additional file 1: Table S4.** Selection of localities of present occurrence and number of potential generations per year.

| Town | Federal State | Latitude | Longitude | Coordinates | Reference | Number of potential generations per year | | |
| --- | --- | --- | --- | --- | --- | --- | --- | --- |
|  |  |  |  |  |  | Present | Future, low CO_2_ | Future, high CO_2_ |
| Unknown | Baden-Württemberg | 47.8197 | 7.5656 | Inferred by georeferencing Fig. 1 | Becker et al. 2011^1^ | 5.45 | 6.29 | 6.69 |
| Baiersbronn | Baden-Württemberg | 48.5071 | 8.3761 | Taken from Table 1 | Huber et al. 2014^2^ | 4.62 | 5.35 | 5.58 |
| Bonn-Holtorf | North-Rhine-Westphalia | 50.7295 | 7.1838 | Taken from Table 1 | Melaun et al. 2015^3^ | 5.14 | 5.91 | 6.16 |
| Unknown | Lower Saxony | 52.2794 | 9.8872 | Inferred by georeferencing Fig. 2 | Werner & Kampen 2013^4^ | 4.80 | 5.59 | 5.91 |
| Leutesdorf | Rheinland-Palatinate | 50.4523 | 7.3863 | Taken from Table 1 | Melaun et al. 2015^3^ | 5.39 | 5.94 | 6.22 |
| Biberach (Baden) | Baden-Württemberg | 48.3483 | 8.0341 | This study, Bock et al. 2015 | This study, Bock et al. 2015^5^ | 5.60 | 6.35 | 6.70 |

^1^Becker N, Huber K, Pluskota B, Kaiser A. *Ochlerotatus japonicus japonicus* – a newly established neozoan in Germany and a revised list of the German mosquito fauna. European Mosquito Bulletin 2011;29:88-102. ^2^Huber K, Schuldt K, Rudolf M, Marklewitz M, Fonseca DM, Kaufmann C, Tsuda Y, Junglen S, Krüger A, Becker N, Tannich E, Becker SC. Distribution and genetic structure of *Aedes japonicus japonicus* (Diptera: Culicidae) in Germany. Parasitology Research 2014;113:3201-3210. Doi: 10.1007/s00436-014-4000-z. ^3^Melaun C, Werblow A, Cunze S, Zotzmann S, Koch LK, Mehlhorn H, Dörge DD, Huner K, Tackenberg O, Klimpel S. Modeling of the putative distribution of the arbovirus vector *Ochlerotatus japonicus japoniucs* (Diptera: Culicidae) in Germany. Parasitology Research 2015;114:1051-1061. Doi: 10.1007/s00436-014-4274-1. ^4^Werner D, Kampen H. The further spread of *Aedes japonicus japonicus* (Diptera, Culicidae) towards northern Germany. Parasitology Research 2013;112:3665-3668. Doi: 10.1007/s00436-013-3564-3. ^5^Bock F, Kuch U, Pfenninger M, Müller R. Standardized laboratory feeding of larval *Aedes japonicus japonicus* (Diptera: Culicidae). Journal of Insect Science 2015;15(1):144. Doi: 10.1093/jisesa/iev126.

**Additional file 1: Table S5.** Test statistics for sex ratio tests.

| Exact binominal test of sex ratio, two-sided* | | | |
| --- | --- | --- | --- |
| Temperature [°C] | Females (total) | Success probability | P value |
| 0ǁ | 0 (0) | NA | NA |
| 5ǁ | 0 (1 larva) | NA | NA |
| 10 | 87 (168) | 0.5179 | 0.6998 |
| 12 | 65 (124) | 0.5242 | 0.6536 |
| 14 | 76 (164) | 0.4634 | 0.3904 |
| 15 | 101 (170) | 0.5941 | **0.0172** |
| 17 | 94 (162) | 0.5802 | 0.0492 |
| 19 | 76 (141) | 0.5390 | 0.3998 |
| 20 | 176 (354) | 0.4972 | 0.9576 |
| 23 | 62 (104) | 0.5962 | 0.0619 |
| 25 | 179 (345) | 0.5188 | 0.5183 |
| 26 | 107 (188) | 0.5691 | 0.0680 |
| 27 | 99 (234) | 0.4231 | **0.0219** |
| 28 | 98 (175) | 0.5600 | 0.1303 |
| 29 | 53 (117) | 0.4530 | 0.3553 |
| 31 | 23 (25) | 0.9200 | **<0.0001** |

ǁ This temperature treatment was not included into the analyses due to the high observed mortality (Tab. 1). * The hypothesised success probability of the binominal test was 0.5. df – degrees of freedom. NA – not analysed. Bold p values are significant (<0.03).

**Additional file 1: Table S6.** R1 wing vein length measurements.

| Temperature [°C] | R1 wing vein length [µm] | | |
| --- | --- | --- | --- |
|  | Mean | sd | N |
| Males | | | |
| 10 | 3332.82 | 105.39 | 66 |
| 12 | 3282.65 | 208.65 | 36 |
| 14 | 3223.23 | 132.78 | 59 |
| 15 | 3317.07 | 102.87 | 57 |
| 17 | 3092.48 | 77.37 | 52 |
| 19 | 3127.60 | 110.91 | 42 |
| 20 | 2999.80 | 142.96 | 63 |
| 23 | 2835.15 | 58.57 | 22 |
| 25 | 2863.90 | 112.98 | 50 |
| 26 | 2739.74 | 88.62 | 67 |
| 27 | 2747.54 | 104.19 | 118 |
| 28 | 2769.86 | 100.26 | 64 |
| 29 | 2623.11 | 91.07 | 54 |
| 31 | 2561.87 | 9.55 | 2 |
| Females | | | |
| 10 | 4183.87 | 142.40 | 76 |
| 12 | 4249.41 | 125.91 | 49 |
| 14 | 4157.70 | 126.78 | 62 |
| 15 | 4260.84 | 173.03 | 70 |
| 17 | 3975.15 | 96.66 | 72 |
| 19 | 4013.49 | 168.63 | 45 |
| 20 | 3926.07 | 160.91 | 55 |
| 23 | 3663.85 | 288.30 | 32 |
| 25 | 3692.36 | 158.11 | 118 |
| 26 | 3494.69 | 103.00 | 97 |
| 27 | 3416.04 | 166.51 | 81 |
| 28 | 3434.57 | 151.37 | 54 |
| 29 | 3182.84 | 117.84 | 47 |
| 31 | 3039.69 | 115.32 | 20 |


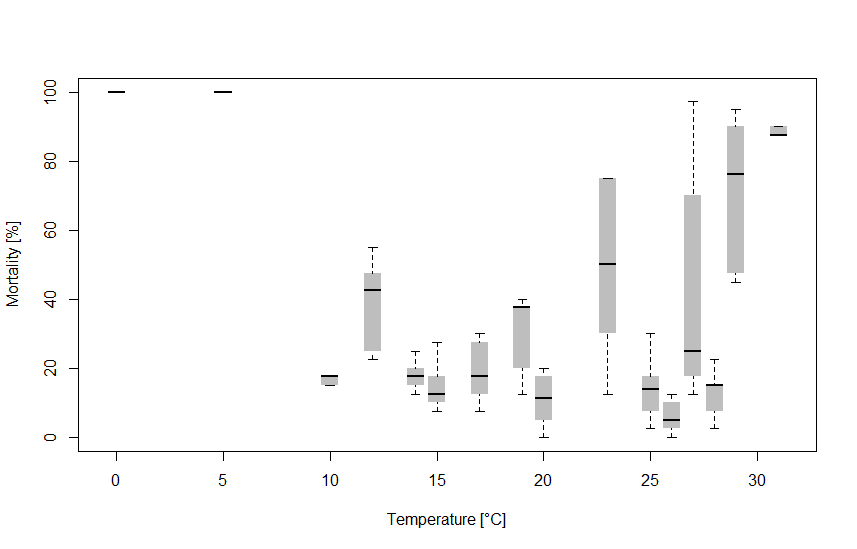


**Additional file 1: Figure S1.** Median mortality per temperature. Boxplots show quartile ranges and medians and whiskers depict 1.5 times the interquartile range. Mortality was calculated considering individuals, which did not survive to emergence. Mortality of the 5°C experiment was assessed on day 113 (see Table 1).


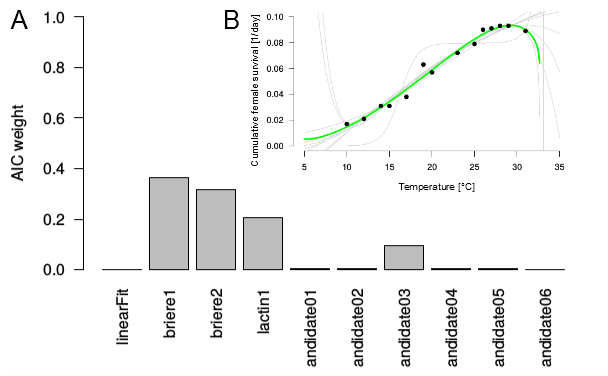


**Additional file 1: Figure S2.** Thermal performance: results of model selection. Model selection using the github R package *thermPerf*^6^ with the cumulative larval survival as performance variable. A: The importance of each model is shown as AICc weights with low weights showing models better fitting to the data. B: Fits of all ten models with the best fit as green line.

During model selection, the empirical constants were calculated to a=0.00006 and m=2.127 for Briere et al. [46] eq. 2. Since m=2 is implemented in Briere et al. [46] eq. 1 and since this model was considered best for fitting the data, non-linear regression of temperature-dependent cumulative female survival was done with this model (R^2^_adj_=0.68) in order to calculate minimum, optimum and maximum temperatures for development.

^6^Bruneaux M. thermPerf: Model fitting for thermal performance curves. NA. R package version 0.0.1. https://github.com/mdjbru-R-packages/thermPerf. Accessed 31 Jul 2017.


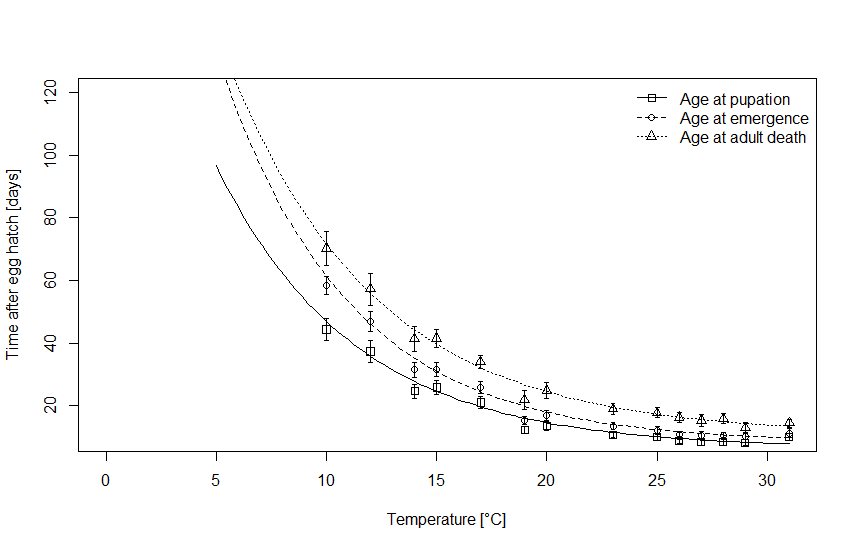


**Additional file 1: Figure S3.** Life trait parameters as functions of temperature. Exponential curves fitted to the life cycle parameters age at pupation, age at emergence and age at adult death. The curve parameters are given in Table S3.
